# Rheumatoid arthritis and excess mortality: down but not out. A primary care cohort study using data from Clinical Practice Research Datalink

**DOI:** 10.1093/rheumatology/key013

**Published:** 2018-02-23

**Authors:** Abhishek Abhishek, Georgina Nakafero, Chang-Fu Kuo, Christian Mallen, Weiya Zhang, Matthew J Grainge, Michael Doherty

**Affiliations:** 1 Academic Rheumatology, Division of Rheumatology, Orthopaedics, and Dermatology, School of Medicine, University of Nottingham, Nottingham, UK; 2 Division of Rheumatology, Allergy and Immunology, Chang Gung Memorial Hospital, Taoyuan, Taiwan; 3 Arthritis Research UK Primary Care Centre, Keele University, Keele, UK; 4 Division of Epidemiology and Public Health, School of Medicine, University of Nottingham, Nottingham, UK

**Keywords:** rheumatoid arthritis, mortality, temporal trends

## Abstract

**Objectives:**

To examine temporal trends in all-cause and cause-specific mortality in RA.

**Methods:**

Data from the Clinical Practice Research Datalink were used. Incident RA cases and four age-, sex- and general practice–matched controls were identified from at-risk cohorts for each calendar year and followed-up for up to 5 years. Mortality rates and 95% CIs were computed. Cox proportional hazard ratios (HRs) were calculated to estimate associations and adjusted for covariates. The temporal trend in mortality was examined using the Joinpoint regression program. Data management and analysis were performed using Stata version 14.

**Results:**

A total of 21 622 cases with incident RA and 86 488 controls were included. The mortality rate of RA cases and controls was 26.90 (95% CI 25.87, 27.97) and 18.92 (18.48, 19.36)/1000 person-years, respectively. The mortality rate in RA cases did not change significantly between 1990 and 2004 but decreased by 7.7%/year between 2005 and 2009. However, the mortality rate in controls improved steadily by 2.2%/year between 1990 and 2009. RA was associated with a 32% excess risk of mortality in the entire cohort [adjusted HR 1.32 (95% CI 1.26, 1.38)], but this was only 15% in cases incident after 2006 [adjusted HR 1.15 (95% CI 1.03, 1.29)]. Similarly, the HR of death due to cardiovascular diseases decreased in cases incident in recent years.

**Conclusion:**

The mortality rate in RA cases incident after the year 2006 has declined significantly, with a trend towards a decline in death from cardiovascular diseases. This could be due to improved management of RA. However, even in cohorts from recent years, RA still associates with higher mortality rates.

Rheumatology key messages

- Rheumatoid arthritis associates with excess mortality.
- The mortality rate of incident RA has decreased over time, with a significant reduction between 2004 and 2009.
- The risk of death due to cardiovascular causes has decreased in people with RA in recent years.

## Introduction

RA is traditionally believed to confer a 50–60% higher risk of mortality [1–3]. However, a recent study from Canada reported that RA cases incident between the years 2001 and 2006 had similar mortality as matched controls, while another study using data from UK’s Health Improvement Network reported elevated but improving mortality in RA [4, 5]. Contrary to these studies, others report that the excess mortality risk associated with RA has not improved in cases incident in recent years [2, 6–10]. As mortality is the most definitive and significant outcome, it is important to confirm if the excess risk of mortality associated with RA has improved in cases incident in recent years who have access to better treatment options. Thus the objectives of this study were to examine the temporal trends in all-cause and cause-specific mortality among adults with incident RA according to the calendar year of disease onset.

## Methods

Data from the Clinical Practice Research Datalink (CPRD) were used in this study. The CPRD is one of the largest databases of longitudinal primary care medical records. Established in 1987, it contains anonymized health care records from >13 million individuals and represents 8% of the UK population at any time. The data in the CPRD undergo thorough quality checks and are of a reliable research standard, with a median 89% with confirmed diagnosis in validation studies [11].

This study was approved by the Independent Scientific Advisory Committee of the Medicines and Healthcare Products Regulatory Agency (15_101R) and included participants contributing acceptable quality data between 1 January 1990 and 31 December 2014. Incident cases with RA were identified from at-risk cohorts for each calendar year. The at-risk cohorts comprised all individuals registered with up-to-standard practices during the year specified who were never coded as having RA before the latest of the current registration date plus 365 days or 1 January of the calendar year specified and were >18 years of age on 1 January of that year. To be eligible as an incident case, participants had to have at least 1 year disease-free registration prior to the first date of RA diagnosis, as this reduces the risk of prevalent cases being counted as incident cases [12]. RA was defined using 58 Read codes (supplementary Table S1, available at *Rheumatology* online) [1, 13] and 93.1% of these included RA or described complications. Four codes implying juvenile arthritis were included, as RA can be misdiagnosed as juvenile arthritis in individuals >17 years of age.

Four controls matched for age (5 year age band), sex and general practice (GP) surgery were randomly selected from individuals without RA for each incident case from the at-risk cohorts in that year. Each matched control was allocated the index case’s date of RA diagnosis. Each case and matched control was followed up for a maximum 5 years from the index date to allow equal follow-up. Person-years of follow-up were calculated from the index date to the earliest of the transfer out date, last data collection date, death date or 5 years from the index date. Data for the cause of death (1 January 1998 onwards), classified according to the International Classification of Diseases, 10th revision, and provided by the Office for National Statistics via CPRD linkage was used to examine temporal trends in cause of death.

### Statistical analysis

Mean (s.d.) and *n* (%) were used for descriptive statistics. Mortality rates and 95% CIs were computed. The Cox proportional hazard ratio (HR) was used to estimate the association between RA and mortality in each at-risk cohort constructed for individual calendar years. This was adjusted for BMI, smoking status, alcohol intake and Charlson comorbidity index (excluding codes for RA) at the start of that year [14, 15]. The temporal trend in risk of mortality was examined using the Joinpoint regression program [16]. This uses Bayesian information criterion to generate different numbers of joinpoints indicating time points where the rate or hazard of occurrence of an event changes significantly and to fit separate linear trends in each time segment. Annual percentage changes (APCs) for each segment were calculated. Competing risk analysis was used to examine the association between RA and death due to cardiovascular, neoplastic and respiratory causes, as they account for most of the deaths in RA [17]. This was stratified for the year of incidence of RA and the APC was calculated as above. Data management and analysis were performed using Stata version 14 (StataCorp, College Station, TX, USA). The statistical significance level was *P* < 0.05.

## Results

Data for 21 622 cases with incident RA and 86 488 matched controls followed up for a mean of 4.31 (s.d. 1.27) and 4.37 (s.d. 1.23) years and contributing 93 122 and 378 002 person-years of data, respectively, were included (supplementary Table S2, available at *Rheumatology* online). There were 2505 (11.59%) and 7150 (8.27%) deaths at a mean age of 77.33 (s.d. 10.56) and 79.09 (s.d. 10.28) years in cases and controls, respectively (*P* < 0.001).

The mortality rates of RA cases and matched controls were 26.90 (95% CI 25.87, 27.97) and 18.92 (18.48, 19.36)/1000 person-years, respectively. RA associated with a higher risk of all-cause mortality [HR 1.42 (95% CI 1.36, 1.49) overall, 1.43 (1.33, 1.54) for men and 1.42 (1.34, 1.50) for women], which remained significant after adjusting for covariates [adjusted HR 1.32 (95% CI 1.26, 1.38) overall, 1.31 (1.21, 1.41) for men, 1.33 (1.25, 1.41) for women].

RA cases and matched controls from recent calendar years demonstrated lower all-cause mortality than that observed in earlier years (supplementary Fig. S1, available at *Rheumatology* online). On joinpoint analysis the 5 year mortality rate for RA cases did not change between 1990 and 2004, but decreased significantly by 7.7%/year (95% CI 1.9, 13.3) between 2004 and 2009, with a statistically significant joinpoint in the year 2004 (supplementary Fig. S2, available at *Rheumatology* online). In contrast, the 5 year mortality rates for matched controls decreased by 2.2%/year (95% CI 3, 1.3) between 1990 and 2009, with no statistically significant joinpoint.

Similar to the improvements in all-cause mortality rate, the HR of mortality associated with RA in recent years has improved (Table 1). Findings from recent years suggest a non-significant increase in the risk of mortality associated with RA. However, this may be due to a lack of power, and when data from 2007 to 2009 were pooled there was a 15% higher risk of mortality in cases incident in these years (Table 2).

**Table 1:** Risk of mortality associated with RA cases incident in each calendar year compared with controls

Year	HR (95% CI)^a^	Adjusted HR (95% CI)^b^
1990	1.48 (1.15, 1.91)	1.30 (1.01, 1.67)
1991	1.57 (1.19, 2.07)	1.53 (1.15, 2.02)
1992	1.15 (0.86, 1.52)	1.21 (0.91, 1.61)
1993	1.49 (1.14, 1.94)	1.53 (1.17, 2.00)
1994	1.45 (1.11, 1.91)	1.51 (1.15¸1.99)
1995	1.28 (0.95, 1.71)	1.23 (0.91¸1.66)
1996	1.17 (0.89, 1.55)	1.19 (0.90¸1.57)
1997	1.40 (1.11, 1.76)	1.45 (1.15, 1.82)
1998	1.40 (1.11, 1.78)	1.44 (1.13, 1.83)
1999	1.43 (1.16, 1.75)	1.31 (1.07, 1.62)
2000	1.65 (1.39, 1.95)	1.58 (1.32, 1.88)
2001	1.68 (1.42, 1.98)	1.69 (1.43, 1.99)
2002	1.51 (1.29, 1.77)	1.48 (1.26, 1.73)
2003	1.36 (1.16, 1.60)	1.25 (1.06, 1.48)
2004	1.54 (1.33¸1.78)	1.42 (1.22, 1.65)
2005	1.35 (1.12, 1.62)	1.22 (1.01, 1.46)
2006	1.40 (1.18, 1.67)	1.27 (1.06, 1.51)
2007	1.31 (1.08, 1.58)	1.14 (0.94, 1.38)
2008	1.21 (1.01, 1.46)	1.11(0.92, 1.33)
2009	1.35 (1.10¸1.66)	1.18 (0.96, 1.45)

a Age, sex and GP surgery matched.

b Matched analysis adjusted for BMI (<20, 20–24.99, 25–29.99, 30–39.99, ≥40 kg/m^2^, missing), smoking status (current smoker, ex-smoker, non-smoker, missing data), alcohol intake (currently, previously, never, missing data) and Charlson comorbidity index (0, 1, ≥2).

**Table 2:** Risk of all-cause and cause-specific mortality associated with RA cases incident in each period

Year	Adjusted HR (95% CI)^a^^,b^			
	All cause	Cardiovascular	Neoplastic	Respiratory
1990–2009	1.32 (1.26, 1.38)	1.23 (1.10, 1.38)^c^	1.13 (1.01, 1.29)^c^	1.43 (1.21, 1.68)^c^
1990–91	1.39 (1.15, 1.68)	—	—	—
1992–94	1.42 (1.21, 1.67)	—	—	—
1995–97	1.32 (1.13, 1.53)	—	—	—
1998–2000	1.45 (1.29, 1.63)	1.42 (1.10, 1.84)	1.34 (1.01¸1.77)	1.38 (0.92, 2.07)
2001–03	1.46 (1.32, 1.60)	1.23 (1.01, 1.49)	1.03 (0.81, 1.29)	1.49 (1.10, 2.04)
2004–06	1.32 (1.20, 145)	1.13 (0.91, 1.40)	1.19 (0.94, 1.50)	1.44 (1.05, 1.97)
2007–09	1.15 (1.03, 1.29)	1.19 (0.92, 1.54)	1.04 (0.80, 1.37)	1.34 (0.97, 1.86)

a Age, sex and GP surgery matched.

b Adjusted for BMI (<20, 20–24.99, 25–29.99, 30–39.99, ≥40 kg/m^2^, missing data), smoking status (current smoker, ex-smoker, non-smoker, missing data), alcohol intake (currently, previously, never, missing data) and Charlson comorbidity index (0, 1, ≥2).

c 1998–2009 for cause-specific mortality.

Data for cause of death were available for 53 949 participants (10 841 cases, 43 108 controls) with index date 1 January 1998–31 December 2009. RA associated with an elevated risk of death from cardiovascular, respiratory and neoplastic causes (Table 2). There was a statistically non-significant trend towards a decline in the risk of death due to cardiovascular causes [APC −3.6 (95% CI −8.1, 1.1), *P* = 0.10] in this period, while the risk of death due to respiratory and neoplastic causes remained stable [APC −1.8 (95% CI −7.9, 4.0), *P* = 0.5; −1.2 (−6.7, 4.6), *P* = 0.7, respectively].

## Discussion

This study demonstrates that the increased risk of death associated with RA has decreased in cases incident after the year 2004 but is still higher than in matched controls. We built separate incidence cohorts for each year that allowed us to examine the effects of treatment paradigm in the calendar and subsequent years on the mortality risk of cases incident in that year. This is a significant advance on previous studies that merged cases from several years into two distinct cohorts [4, 5]. Additionally, the findings of this study have greater validity than previous studies, as we included a longer study period (1990–2014), had contemporaneous matched controls and used competing risk analysis when estimating the risk of cause-specific mortality. Moreover, joinpoint analysis allows us to identify the year in which excess mortality associated with RA began to decrease.

The mortality rates reported in this study are consistent with previous reports [5, 8, 10]. We observed a steady decline in mortality in controls between the years 1990 and 2014, whereas there was a significant reduction in the risk of death in RA cases incident after the year 2004. This suggests that the adoption of early combination DMARD treatment, supported by biologics when indicated, may have saved lives of RA cases incident in recent years [18, 19].

As previously reported, we observed a trend towards a reduction in the risk of death from cardiovascular diseases [4]. However, the elevated risk of death due to respiratory diseases and neoplasms appeared to be stable in this study, in contrast to previous reports [4]. This raises the possibility that improved control of inflammation in RA with the availability of better treatment options has translated into reduced cardiovascular mortality, potentially via an effect on stabilizing the progression of atherosclerosis [20], but has not reduced mortality risk due to neoplasms and respiratory causes.

A previous study using data from the Health Improvement Network reported that RA cases incident between 1999 and 2006 and 2007 and 2014 had 56 and 29% higher risk of death, respectively [5]. This is higher than the overall mortality risk reported in our study. We found RA cases incident between 2007 and 2009 to only have a 15% excess mortality risk, which is lower than the risk of mortality (cf. 32–46%) in cases incident in earlier years. Our study with a longer period of follow-up allows an independent corroboration of the previous studies examining temporal trends in RA mortality.

The improvement in mortality in people with RA is not due to screening for cardiovascular diseases, as this was included in the Quality and Outcomes Framework, a mechanism by which GP surgeries were rewarded financially for taking better care of people with certain conditions in the year 2014. It is possible that this improvement in mortality is due to the availability of anti-CCP antibody testing in the UK, which would result in a greater proportion of people with inflammatory arthritis being diagnosed with RA and receiving combination DMARDs.

In this study we used 1 year disease-free up to standard registration in the CPRD to define incident RA cases, because the incidence of RA does not vary when increasing this to 3 years [1]. Additionally, we used the first Read code for RA to define incident RA. Others have used one Read code of an RA diagnosis and one DMARD prescription to define cases as having RA. We believe that the latter method may induce ascertainment bias in defining incident RA cases in a study spanning >20 years during which DMARD prescribing practices have changed, with a >100% increase in the uptake of DMARDs over time. Therefore, selecting cases with RA based on at least one prescription for a DMARD would result in only more severe cases being included in earlier cohorts, while the latest cohorts could have the full spectrum of RA cases, both mild and severe. Such an inclusion criteria would bias the study towards showing a temporal trend in mortality reduction.

There are several caveats to this study. First, RA is diagnosed by hospital rheumatologists in the UK and the coding in the CPRD is likely to lag behind the diagnosis by several weeks due to the time taken for clinic letters to arrive in GP surgeries. Thus some cases coded as having RA in 1 year may have been diagnosed in the previous year. However, this is unlikely to play a significant role, as misclassification from consecutive calendar years is likely to cancel out effects on the temporal trend. Second, the CPRD is a consultation-based database and comorbidities are recorded only if a patient consults for them. Finally, we are unable to provide a reason for the reduction in mortality in RA and are only able to speculate that this is due to improved management of RA in recent years. However, given the degree of improvement in RA mortality and changes in the paradigm of treatment of RA in the early 2000s, this seems likely. The CPRD is limited by the absence of detailed phenotyping of RA cases, for example, information on affected joints is not recorded. We are therefore unable to specify the proportion of cases who meet the 2010 ACR/EULAR classification criteria for RA. Another limitation is that we examined mortality in the first 5 years of disease, and further research is required to understand if this trend persists in the long term.

In conclusion, the risk of mortality due to RA improved after 2004, with a continuing decline in recent years, and is likely to be attributed to improving cardiovascular mortality.

## Supplementary Material

Supplementary Data

## References

[1] Abhishek A, Doherty M, Kuo C-F Rheumatoid arthritis is getting less frequent—results of a nationwide population-based cohort study. Rheumatology 2017;56:736–44. 28064207 10.1093/rheumatology/kew468 PMC5850292

[2] Dadoun S, Zeboulon-Ktorza N, Combescure C Mortality in rheumatoid arthritis over the last fifty years: systematic review and meta-analysis. Joint Bone Spine 2013;80:29–33. 22459416 10.1016/j.jbspin.2012.02.005

[3] Sokka T, Abelson B, Pincus T. Mortality in rheumatoid arthritis: 2008 update. Clin Exp Rheumatol 2008;26(5 Suppl 51):S35–61. 19026144

[4] Lacaille D, Avina-Zubieta JA, Sayre EC, Abrahamowicz M. Improvement in 5-year mortality in incident rheumatoid arthritis compared with the general population-closing the mortality gap. Ann Rheum Dis 2017;76:1057–63. 28031164 10.1136/annrheumdis-2016-209562 PMC5526676

[5] Zhang Y, Lu N, Peloquin C Improved survival in rheumatoid arthritis: a general population-based cohort study. Ann Rheum Dis 2017;76:408–13. 27338777 10.1136/annrheumdis-2015-209058 PMC5182185

[6] Edwards CJ, Campbell J, van Staa T, Arden NK. Regional and temporal variation in the treatment of rheumatoid arthritis across the UK: a descriptive register-based cohort study. BMJ Open 2012;2:e001603. 10.1136/bmjopen-2012-001603 PMC3533005 23144258

[7] Widdifield J, Bernatsky S, Paterson JM Trends in excess mortality among patients with rheumatoid arthritis in Ontario, Canada. Arthritis Care Res 2015;67:1047–53. 10.1002/acr.22553 25623141

[8] Gonzalez A, Maradit Kremers H, Crowson CS The widening mortality gap between rheumatoid arthritis patients and the general population. Arthritis Rheum 2007;56:3583–7. 17968923 10.1002/art.22979

[9] Radovits BJ, Fransen J, Al Shamma S Excess mortality emerges after 10 years in an inception cohort of early rheumatoid arthritis. Arthritis Care Res 2010;62:362–70. 10.1002/acr.20105 20391482

[10] Humphreys JH, Warner A, Chipping J Mortality trends in patients with early rheumatoid arthritis over 20 years: results from the Norfolk Arthritis Register. Arthritis Care Res 2014;66:1296–301. 10.1002/acr.22296 PMC4226330 24497371

[11] Herrett E, Thomas SL, Schoonen WM, Smeeth L, Hall AJ. Validation and validity of diagnoses in the General Practice Research Database: a systematic review. Br J Clin Pharmacol 2010;69:4–14. 20078607 10.1111/j.1365-2125.2009.03537.x PMC2805870

[12] Lewis JD, Bilker WB, Weinstein RB, Strom BL. The relationship between time since registration and measured incidence rates in the General Practice Research Database. Pharmacoepidemiol Drug Saf 2005;14:443–51. 15898131 10.1002/pds.1115

[13] Nicholson A, Ford E, Davies KA Optimising use of electronic health records to describe the presentation of rheumatoid arthritis in primary care: a strategy for developing code lists. PLoS One 2013;8:e54878. 23451024 10.1371/journal.pone.0054878 PMC3579840

[14] Khan NF, Perera R, Harper S, Rose PW. Adaptation and validation of the Charlson Index for Read/OXMIS coded databases. BMC Fam Pract 2010;11:1. 20051110 10.1186/1471-2296-11-1 PMC2820468

[15] Charlson M, Szatrowski TP, Peterson J, Gold J. Validation of a combined comorbidity index. J Clin Epidemiol 1994;47:1245–51. 7722560 10.1016/0895-4356(94)90129-5

[16] Kim HJ, Fay MP, Feuer EJ, Midthune DN. Permutation tests for joinpoint regression with applications to cancer rates. Stats Med 2000;19:335–51. 10.1002/(sici)1097-0258(20000215)19:3<335::aid-sim336>3.0.co;2-z 10649300

[17] Movahedi M, Costello R, Lunt M Oral glucocorticoid therapy and all-cause and cause-specific mortality in patients with rheumatoid arthritis: a retrospective cohort study. Eur J Epidemiol 2016;31:1045–55. 27256352 10.1007/s10654-016-0167-1 PMC5065607

[18] Luqmani R, Hennell S, Estrach C British Society for Rheumatology and British Health Professionals in Rheumatology guideline for the management of rheumatoid arthritis (the first two years). Rheumatology 2006;45:1167–9. 16844700 10.1093/rheumatology/kel215a

[19] Saag KG, Teng GG, Patkar NM American College of Rheumatology 2008 recommendations for the use of nonbiologic and biologic disease-modifying antirheumatic drugs in rheumatoid arthritis. Arthritis Rheum 2008;59:762–84. 18512708 10.1002/art.23721

[20] Hansson GK. Inflammation, atherosclerosis, and coronary artery disease. N Engl J Med 2005;352:1685–95. 15843671 10.1056/NEJMra043430

