# Supplementary material for: Rheumatoid arthritis and excess mortality: down but not out. A primary care cohort study using data from Clinical Practice Research Datalink
Source: Rheumatology (Oxford). 2018 Feb 23;57(6):977–81. doi: 10.1093/rheumatology/key013 (PMC5965085; doi:10.1093/rheumatology/key013)
Supplement: Supplementary Data [file key013_rhe-17-1501-file002.docx]

**SUPPLEMENTARY DATA**

**Supplementary Table S1: RA Codes used to define cases in this study**

| **Read Code** | **Read Term** |
| --- | --- |
| N040.00 | Rheumatoid arthritis |
| N043.00 | Juvenile rheumatoid arthritis - Still's disease |
| N042200 | Rheumatoid nodule |
| N040P00 | Seronegative rheumatoid arthritis |
| N045.00 | Other juvenile arthritis |
| N040T00 | Flare of rheumatoid arthritis |
| N047.00 | Seropositive errosive rheumatoid arthritis |
| H570.00 | Rheumatoid lung |
| N04X.00 | Seropositive rheumatoid arthritis, unspecified |
| 66H..13 | Rheumatoid arthrit. monitoring |
| N040Q00 | Rheumatoid bursitis |
| N040200 | Rheumatoid arthritis of shoulder |
| N043200 | Pauciarticular juvenile rheumatoid arthritis |
| N362200 | Swan-neck finger deformity |
| N041.00 | Felty's syndrome |
| N043z00 | Juvenile rheumatoid arthritis NOS |
| N04..00 | Rheumatoid arthritis and other inflammatory polyarthropathy |
| N04y012 | Fibrosing alveolitis associated with rheumatoid arthritis |
| N040N00 | Rheumatoid vasculitis |
| N040S00 | Rheumatoid arthritis - multiple joint |
| N045100 | Juvenile seronegative polyarthritis |
| F396400 | Myopathy due to rheumatoid arthritis |
| N045500 | Juvenile rheumatoid arthritis |
| N04y000 | Rheumatoid lung |
| 2G27.00 | O/E-hands-rheumatoid spindling |
| N043300 | Monarticular juvenile rheumatoid arthritis |
| 2G25.11 | O/E - ulnar deviation |
| N042z00 | Rheumatoid arthropathy + visceral/systemic involvement NOS |
| N040900 | Rheumatoid arthritis of PIP joint of finger |
| N040800 | Rheumatoid arthritis of MCP joint |
| G5yA.00 | Rheumatoid carditis |
| N040100 | Other rheumatoid arthritis of spine |
| N040000 | Rheumatoid arthritis of cervical spine |
| N042100 | Rheumatoid lung disease |
| N045600 | Pauciarticular onset juvenile chronic arthritis |
| N043100 | Acute polyarticular juvenile rheumatoid arthritis |
| N040700 | Rheumatoid arthritis of wrist |
| N040B00 | Rheumatoid arthritis of hip |
| N042.00 | Other rheumatoid arthropathy + visceral/systemic involvement |
| G5y8.00 | Rheumatoid myocarditis |
| N043000 | Juvenile rheumatoid arthropathy unspecified |
| N040D00 | Rheumatoid arthritis of knee |
| N040K00 | Rheumatoid arthritis of 1st MTP joint |
| N040F00 | Rheumatoid arthritis of ankle |
| N040R00 | Rheumatoid nodule |
| Nyu1G00 | [X]Seropositive rheumatoid arthritis, unspecified |
| 2G25.00 | O/E - hands - ulnar deviation |
| N04y011 | Caplan's syndrome |
| N040500 | Rheumatoid arthritis of elbow |
| F371200 | Polyneuropathy in rheumatoid arthritis |
| N040A00 | Rheumatoid arthritis of DIP joint of finger |
| N040600 | Rheumatoid arthritis of distal radio-ulnar joint |
| Nyu1200 | [X]Other specified rheumatoid arthritis |
| N040H00 | Rheumatoid arthritis of talonavicular joint |
| N040J00 | Rheumatoid arthritis of other tarsal joint |
| N040G00 | Rheumatoid arthritis of subtalar joint |
| Nyu1100 | [X]Other seropositive rheumatoid arthritis |
| Nyu1500 | [X]Other juvenile arthritis |

**Supplementary Table S2. Disease and demographic characteristics of matched cases and controls**

|  | Controls  (n=86,488) | Cases  (n=21,622) | OR (95% CI) |
| --- | --- | --- | --- |
| Age (years), mean (SD) | 60.53 (15.23) | 60.72 (15.17) | -/- |
| Gender, N, women | 59,196 (68.44) | 14,798 (68.44) | -/- |
| BMI (kg/m^2^) |  |  |  |
| 10-19.99 | 4,476 (5.18) | 1,175 (5.43) | 1.06 (0.99-1.13) |
| 20-24.99 | 27,335 (31.61) | 6,778 (31.35) | 1 |
| 25-29.99 | 26,760 (30.94) | 6,745 (31.20) | 1.02 (0.98-1.06) |
| 30-39.99 | 13,942 (16.12) | 3,888 (17.98) | 1.12 (1.08-1.18) |
| ≥40 | 1,501 (1.74) | 439 (2.03) | 1.18 (1.06-1.32) |
| Missing | 12,474 (14.42) | 2,597 (12.01) | 0.84 (0.80-0.88) |
| Alcohol intake |  | | |
| Current | 58,478 (67.61) | 14,429 (66.73) | 1 |
| None | 15,748 (18.21) | 4,482 (20.73) | 1.15 (1.11-1.20) |
| Previous alcohol intake | 1301 (1.50) | 447 (2.07) | 1.39 (1.25-1.55) |
| Unknown | 10,961 (12.67) | 2,264 (10.47) | 0.83 (0.80-0.88) |
| Cigarette smoking |  | | |
| Never | 48,232 (55.77) | 10,526 (48.68) | 1 |
| Current | 16,917 (19.56) | 5,395 (24.95) | 1.46 (1.41-1.52) |
| Ex | 16,738 (19.35) | 5,001 (23.13) | 1.37 (1.32-1.42) |
| Unknown | 4,566 (3.22) | 696 (4.87) | 0.70 (0.64-0.76) |
| Charlson comorbidity index |  |  |  |
| 0 | 58,745 (67.92) | 12,762 (59.02) | 1 |
| 1 | 15,609 (18.05) | 5,041 (23.31) | 1.49 (1.43-1.54) |
| ≥2 | 12,134 (14.03) | 3,819 (17.66) | 1.45 (1.39-1.51) |

Values stated as n (%) unless otherwise stated.

**Supplementary Figure S1. Temporal trend in mortality rate of incident RA and controls**


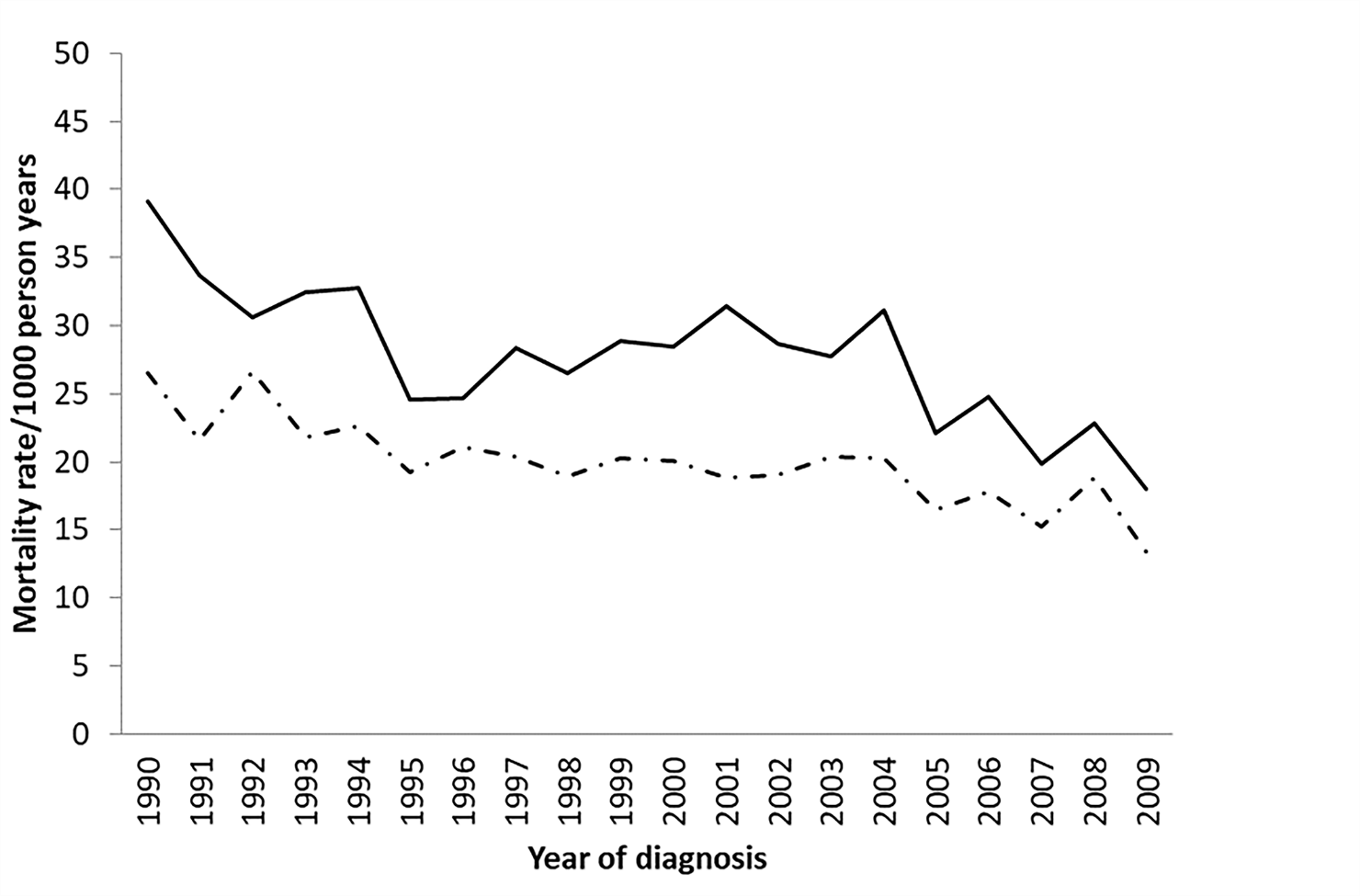


Mortality rate per 1000 person years for cases with incident RA (solid line) in a calendar year and age, sex, and GP surgery matched controls (dashed line) contributing data to CPRD at the time of diagnosis of RA in the index case

**Supplementary Figure S2.** **Joinpoint graph of temporal trend in mortality rate of incident RA and controls**


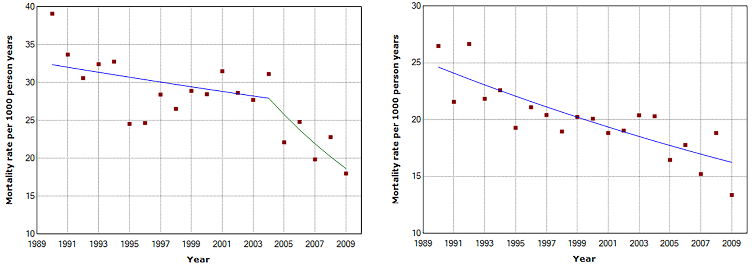


Joinpoint graph (left panel) showing reduction in mortality rate per thousand person years in incident RA cases from cohorts built in subsequent calendar years, with significant join points in the year 2004. Joinpoint graph (right panel) showing progressive reduction in mortality in age, sex and GP surgery matched controls from cohorts built in consecutive calendar years without any significant joinpoints.
